# The bibliometric analysis of scholarly production: How great is the impact?

**DOI:** 10.1007/s11192-015-1645-z

**Published:** 2015-07-28

**Authors:** Ole Ellegaard, Johan A. Wallin

**Affiliations:** Library, University of Southern Denmark, Campusvej 55, Odense M, 5230 Denmark

**Keywords:** Bibliometric analysis, Citation analysis, Publication analysis, Impact of publications

## Abstract

Bibliometric methods or “analysis” are now firmly established as scientific specialties and are an integral part of research evaluation methodology especially within the scientific and applied fields. The methods are used increasingly when studying various aspects of science and also in the way institutions and universities are ranked worldwide. A sufficient number of studies have been completed, and with the resulting literature, it is now possible to analyse the bibliometric method by using its own methodology. The bibliometric literature in this study, which was extracted from Web of Science, is divided into two parts using a method comparable to the method of Jonkers et al. (Characteristics of bibliometrics articles in library and information sciences (LIS) and other journals, pp. 449–551, 2012: The publications either lie within the Information and Library Science (ILS) category or within the non-ILS category which includes more applied, “subject” based studies. The impact in the different groupings is judged by means of citation analysis using normalized data and an almost linear increase can be observed from 1994 onwards in the non-ILS category. The implication for the dissemination and use of the bibliometric methods in the different contexts is discussed. A keyword analysis identifies the most popular subjects covered by bibliometric analysis, and multidisciplinary articles are shown to have the highest impact. A noticeable shift is observed in those countries which contribute to the pool of bibliometric analysis, as well as a self-perpetuating effect in giving and taking references.

## Background

Bibliometric methods have been used for providing quantitative analysis of written publications. Bibliometrics is closely related to the broader term “infometrics” (Egghe and Rousseau 1990; Wolfram 2003) and the narrower term “scientometrics” (Bar-Ilan 2008, 2010). A close analogy is “webometrics”, which examines different aspects of the web. This type of analysis is based on the identification of the corpus of literature, i.e. publications in their broadest sense, within a given subject area. Statistical tools were rapidly used as part of the analysis workset. Originally, it consisted mainly of bibliographic overviews of scientific productions or selections of highly cited publications. These overviews were subdivided into lists of author productions, national or subject bibliographies. Often focus is on a number of broad or more specialized subjects in publishing patterns: It includes geographical (Lin 2012; Zhuang et al. 2013) or institutional aspects, and indicators of performance including development over time periods (Huffman et al. 2013), subject domains or disciplines (Dalpe 2002; Liu et al. 2012; Zibareva et al. 2014) or types of literature and authorships (White and McCain 1998). The analyses encompass various material categories and range from journal articles, books, theses and patents to reports in the category “grey literature”.

In order to extract and manipulate data, bibliometric methods, based on content or citation analysis, are often used (Wallin 2005). These methods have benefited greatly from computerized data treatment and in the recent years there has been a huge increase in the number of publications within the field. This is partly due to the computerized methods but also to the fact that a bibliometric method has to include a certain volume of data in order to be statistically reliable.

Nowadays, a number of new metrics have become available: Download statistics, page ranks, bookmarking tools such as *Mendeley* (Zaugg et al. 2011) and sharing on social media. With all these tools available the impact of scientific literature can be analyzed and interpreted in a multitude of ways. This tendency is further complicated by the growth in non-traditional publishing and the number of scholarly publishing platforms as sources of aggregate data (Meho 2006). There has been an increased focus, not only on quantitative data, but also more generally, on qualitative aspects such as the implications of bibliometric analysis in relation to research evaluation (Herther 2009). The data is interpreted in a number of ways using different variants of the h-index (Hirsch 2005) or similar indexes and via the importance of publishing in high impact-factor journals. The number of co-authored papers and the amount of international collaborations are often taken into account in the evaluation process. Bibliometric methods also play an increasing role in the ranking of research departments and institutions. All these methods are represented in the data set investigated here and focus is placed primarily on the way, the bibliometric methods are received by different user groups.

Content analysis can also provide quantitative measures through harvesting of keywords. Examples are forest ecology research (Song and Zhao 2013) or education and ethics (Marshakova-Shaikevich 2005).This method has the potential of discovering up-and-coming fields. Further, in the literature, analysis of specialized content is possible. Data can be extracted which highlights chemical substances which are hazardous to the public (Grandjean et al. 2011; Ellegaard and Wallin 2013). A recent project has been launched which examines book contents in entirety via analysis of n-grams (Michel et al. 2011). Trends within many areas can be followed over extended periods of time by using this method.

In general, there has been a widespread wish among decision-makers to qualify, respectively to quantify, the research performed. In this context, the bibliometric analysis offers itself naturally as an instrument. But, as already discussed by Glänzel (1996), it is important to be aware of the methods and standards involved in order to get reliable and scalable results. Wallin (2005) thoroughly discuss the pitfalls and possibilities involved in these types of analysis and analyze the impact, visibility or influence the literature has in the scientific community. Citation analysis is probably the most traditional method applied in bibliometrics as an approximate measure of scientific quality particularly in the case of individual researchers, rankings of universities and institutions (Waltman et al. 2012; Weingart 2005) or simply for judging the impact of publications (Frandsen and Rousseau 2005).The method is used increasingly to provide information about interrelations between different groups in the scientific community (Barth et al. 2014).The main reasons for doing these types of analysis can stem from a number of factors and there is a desire within many scientific fields to obtain an overview of the literature. Traditionally, review articles or surveys have provided this. A review article summarizes critically selected scientific content. This content is normally scattered within the literature in combination with an extensive bibliography of the field. In contrast, a bibliometric analysis has its focus on statistical related data but is seldom used in combination with a bibliography of the area. Professionals, who knew their disciplines thoroughly, have produced the review literature and the reports based on a working knowledge of the field. Groups within the same scientific disciplines are the intended audience for these publications. On the other hand, information specialists with special skills often apply the bibliometric methods. External clients order reports or articles and these reports are sometimes made in cooperation with the scientific staff. Institutional or governmental agencies increasingly demand productivity reports or quality assessments of staff performance. Researchers who read these types of analysis become aware of the new trends and competing groups as well as possibilities for cooperation. In all cases, one may use bibliometric methods to advantage.

Nowadays, a number of tools have apparently made it much easier to produce these reports. This ranges from databases such as *Web of Science (WoS), Scopus* or *Google Scholar* (Li et al. 2010) which have added, incorporated reference handling capabilities. *Scival* and *InCites* are sophisticated, analytical tools offered on a commercial basis by the large data base providers as well. In more specialized software e.g. *Gephi* (Bastian et al. 2009), *HistCite* (Garfield 2009), “*Publish or Perish*” (Harzing 2010) or *Scholarometer* (Kaur et al. 2012) a number of different metrics and issues related to normalization procedures can be handled quantitatively (Pellegrino 2011). Indeed, normalization procedures are very important in order to make an analysis based on citation data which makes it possible to compare different groups. A number of alternative metrics has been proposed which could even out differences between field sizes, publication and citation practices (Kaur et al. 2013). These normalized metrics could lend even more credibility to the whole field, especially in cases where the bibliometric methods are applied to the analysis of different disciplines. All these tools may produce, within an ever-increasing number of articles and reports, based on bibliometric methods, a higher level of analysis of research trends, productivity in different fields or scientific connection patterns.

This may raise a question: Do these types of reports serve the intended purpose? Who are in fact consuming and taking advantage of this type of literature? Is there a difference between the approach taken by the professionals or researchers in the many subject fields directly involved (the non-ILS group) and those who participate as information specialists (the ILS-group)? In dealing with these matters, we will apply the very same method: the bibliometric analysis. We will consider publications as well as citation patterns in our documentation and make a clear distinction between the two aforementioned groups; “subject” or “information” professionals who contribute with documentation based on bibliometric methods. This approach follows a method already in use by Jonkers and Derrick (2012) and includes a thorough discussion of the dissemination and interpretation of the bibliometric methods as well as the use of the same methods among the different user groups. ILS and non-ILS bibliometric articles were also categorized, by descriptors of the methods or type of analysis involved, in the work of Derrick et al. (2012).They identified a number of categories in which the publication pattern was different for the two groups. An important finding relates to ‘development and improving of bibliometric methods’ where a significant increase in interest was observed in the non-ILS community. Obviously, this would be of benefit to the work of both communities. These issues have been raised earlier on a general basis with concerns about the bodies of literature evolving separately within the two groups (Glänzel and Schoepflin 1994). Later on, the same types of problems have been raised in the *Leiden Manifesto* by Hicks et al. (2015) that research evaluation is now led by data rather than sound judgement and good practice. The latter is often established through the work of bibliometricians and implemented in cooperation with the user communities.

Furthermore, it is not evident if the growth in number of publications involving bibliometric methods is merely facilitated by the rise in number of publications available for analysis. Most likely, a threshold has to be passed in in order to gain sufficiently statistics but other factors could play a role as well.

An open question is still raised: Is it possible to verify a general shift from basic and methodological research to applied bibliometrics as well as domination of the interests of science policy, as further noticed by Glänzel and Schoepflin (1994)? The use of bibliometric methods is obviously driven by a need to evaluate scientific production and making the results available to policymakers, scientists or other stakeholders. But, one may ask if the rising number of publications is due to a genuine demand for these types of analysis and the investigations serve their intended purpose. Hopefully, the development and distribution over time in the published number of bibliometric analysis and the citation or impact of these within the different fields could indicate the trends and provide the needed answers. We analyze development over time of the impact within both the ILS and non-ILS community and hope to uncover any change in the way they are received by their audience. This could be further substantiated by considering the subjects which are analyzed, as well as identifying the contributions from the more established and up-and-coming countries participating in the field. We try to determine to which degree the ILS and non-ILS communities are involved in the subject-based types of analysis and consider to which extent they operate in separate or overlapping spheres of interest.

## Method

The main investigation is based on the primary literature, mostly scholarly articles, indexed in the major bibliographic databases. Only literature about natural sciences, technical sciences and health sciences including medicine is considered in the present study. The scientific processes, as well as the methods for dissemination of information, are very similar within these fields. The humanities and social sciences have, to a large degree, other types of publication channels and are not included. The database chosen is *WoS* which has the oldest and most comprehensive records of citation indexes and includes a useful analysis tool. *WoS* does not necessarily index the largest number of journals in all the different fields compared to i.e. *Scopus* (Li et al. 2010), but it is assumed that a sufficient amount of high quality literature, especially in the case of medicine and the natural science, can be examined using this database, and all the trends needed to be investigated are properly represented. The research spans 50 years of scientific literature and covers, in practice, the total time since citation indexing was introduced. In order to create a representative corpus of documents for investigation, one may set up the following search profile in *WoS*:

*TS* = *((“bibliometric analysis”) OR (“bibliometric study”) OR (“citation analysis”) OR (“citation study”) OR (“scientometric study”) OR (“scholarly productivity”) OR (“scientometric analysis”) OR (“publication analysis”) OR (“scholarly impact”) OR (“patent citation”)).*

**Indexes = SCI-EXPANDED, CPCI-S. Timespan = 1964–2013**

This profile, although not exhaustive, produces a comprehensive amount of documents for further treatment. The profile is an extended version of a profile based on a keyword analysis of all subject fields (Jonkers and Derrick 2012). This group also characterized the literature according to author affiliation, but found it difficult to reach a clear separation as bibliometricians are not necessarily affiliated with ILS-departments. Instead, the following approach was used: Firstly, the documents have been separated in two main groups based on the *WoS* categories. These categories reflect the different subject content of the articles and correspond to the journal categories in *Thomson Reuters: Journal Citation Reports*. A group is applied which deals with documents belonging to the “Information and Library Science (ILS)” category and the other documents have been merged in another group: The non-ILS category. The documents within the ILS category encompass both fundamental, theoretical studies of bibliometries and more applied, “subject” orientated studies. Those within the non-ILS category are most likely of the latter type. Therefore, documents belonging to the ILS category are tentatively subdivided into two types of studies by using the method of Derrick et al. (2012). They assigned up to thirteen different codes to the articles by examining the content of either title or abstract. Six of these codes such as ‘Analyses a field or topic’ or ‘Analyses collaboration of networks or author behavior’ have been assigned to the applied, ‘subject’ based studies. The rest is used for the more theoretical and methodical articles on the implication and evaluation of bibliometric methods. In this way, by considering the titles and abstracts in our data material and assigning one primary code, we merge the articles from the main ILS category into two subcategories.

The aim in using this method is to quantify the documents referring to bibliometric analysis as a working tool into the following four tiers for further analysis: Tier 1.The whole ILS category with theoretical or fundamental as well as applied studies of bibliometry. Tier 2. Bibliometric studies of theoretical or methodical fields published within the ILS category. Tier 3. Bibliometric studies of applied subjects published within the ILS category and Tier 4. Bibliometric studies of applied subjects published in the non-ILS category. The division of the literature is summarized in Table 1. The analysis will focus primarily on the two latter groups of applied studies, without pursuing the theoretical studies in detail in the present work.

**Table 1:** Division of articles on bibliometric analysis into different tiers

Tier	Category
Tier 1	Information and Library Science (ILS). All studies
Tier 2	Information and Library Science (ILS). Methodical or theoretical studies
Tier 3	Information and Library Science (ILS). Applied or “Subject” related studies
Tier 4	Non-ILS. Applied or “Subject” related studies^a^

^a^Total number of studies: 198

The groups are examined separately with citation analysis in order to judge their impact. The citing documents can be divided in the same manner into ILS and non-ILS groups based on the *WoS*-category to which they belong. This division ensures that any difference in citation pattern between the communities which publish in the two categories can be revealed. A fixed citation window is applied, which expires at the end of 2013 and it thereby follows the same period as used for the collection of the bibliometric articles. In this way the data gathering of citation data was conducted in a reproducible manner.

We define a normalized impact *I*_*n,*norm_ for a corpus of articles published in a span of years up to year *n* in the following manner:

1 $$I_{{n,\;{\text{norm}}}} = C_{n} /\mathop \sum \limits_{i = 1964}^{n - 1} P_{i}$$

*P*_*i*_ is the number of articles published in year *i* and *C*_*n*_ is the total number of citing articles published throughout the years 1964…*n*. This definition is in line with the general definition of the journal impact factor (*JIF*) in a given year as citation to articles published in a predefined number of preceding years, i.e. as done in case of the 5 year *JIF* factor. In this way, we can follow tendencies in the change of impact of the bibliometric methods over time. This data supplements the numbers obtained for the production of articles during the same period. Obviously, the latter numbers are mainly related to the utilization of the bibliometric methods.

The data is analyzed further within the time domain and investigated for any possible lag in the pattern of publication and citation frequency between the different groups. We look at the role of the different countries which are the main actors in the field and also for changes in publication patterns over time. The articles in tier 4 are distributed among a large number of subject fields: Applied, computer, health, physical, life, multidisciplinary science and a number of minor subject fields not included here. An article is placed in i.e. health science if it is alone indexed in this *WoS* science category. In this way, we obtain mutually exclusive sets of articles for further analysis.

The various subjects are considered in larger detail by extracting keywords from the references. All references from tier 4 are downloaded into the reference handling program *EndNote* to perform the keyword analysis. The raw keyword data is then exported to and counted by a small *Delphi* script. A number of keywords are discarded as being trivial. The frequency of the keywords in the references gives a good indication of the subject fields investigated. In this way, we aim to establish a link between publishing patterns and the impact of bibliometric analysis as an applied tool within the various communities.

## Results

### Characteristics of the publications

In Tables 2 and 3, the main data from the search profile and chosen period 1964–2013 are shown.

**Table 2:** Total numbers of articles on bibliometric analysis published in journals indexed by *Web of Science* (*WoS*) during 1964–2013, overview

Article classified in	Number of articles	Number of journals^a^	Articles per journal	Citations	Citations per article	Citing articles
All categories	2854	1138	2.5	28,874	10.1	15,912
Information and Library Science. All studies	1048	59	17.8	12,485	11.9	6474
Information and Library Science. Methodical or theoretical studies	448	39	11.5	6906	15.4	4166
Information and Library Science. Applied or “Subject” related studies	600	50	12.0	5636	9.4	3692
Non-ILS. Applied or “Subject” related studies	1806	1084	1.7	16,386	9.1	11,372

^a^In a few instances, the articles in either the ILS or the non-ILS category have two journal entries

**Table 3:** Number of articles citing articles on bibliometric analysis published in journals indexed by *WoS* during 1964–2013

Article classified in	Citing article classified in	Number of citing articles	Number of citing journals	Citing articles per journal
Information and Library Science. All studies	Information and Library Science. All studies	3813	174	21.9
	Non-ILS category	2661	1463	1.8
Information and Library Science. Methodical or theoretical studies	Information and Library Science. All studies	2566	137	18.7
	Non-ILS category	1600	1027	1.6
Information and Library Science. Applied or “Subject” related studies	Information and Library Science. All studies	2236	148	15.1
	Non-ILS category	1456	879	1.7
Non-ILS. Applied or “Subject” related studies	Information and Library Science. All studies	1939	145	13.4
	Non-ILS category	9433	3480	2.7

**Fig. 1:**
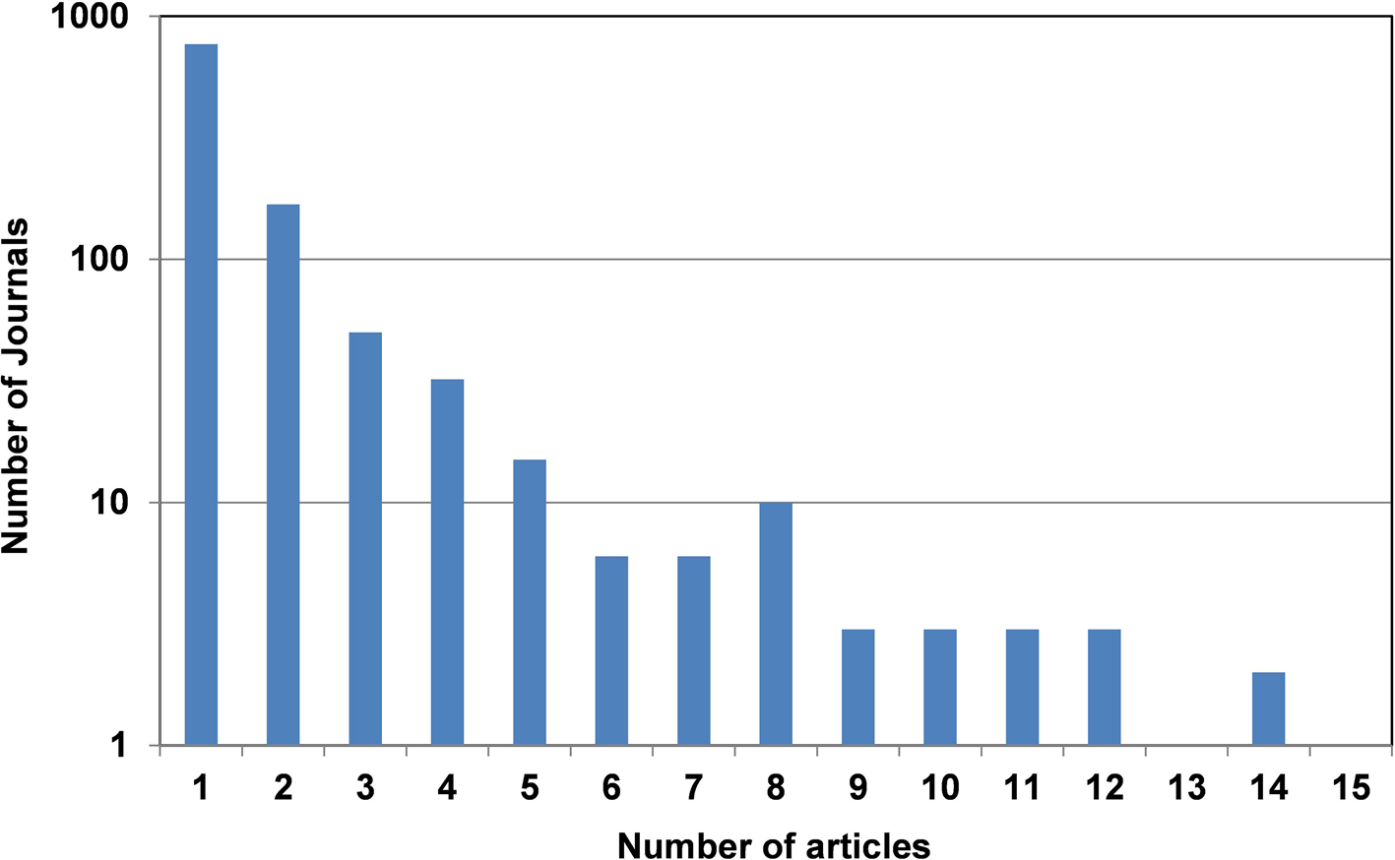
Articles that refer to bibliometric analysis published in *WoS* non-ILS journals. 1964–2013

A significant number of the bibliometric analyse performed (37 %) is classified in the ILS category (tier 1). These articles can be subdivided into methodical, theoretical studies, which are around 43 % (tier 2) and the applied, “subject” based studies accounting for the rest (tier 3).The number of articles in tier 4 (non-ILS category) is almost three times larger than tier 3 (“subject” based types of analysis published in the ILS category) but the articles in tier 4 are scattered among a significantly larger number of journals. This is illustrated in Fig. 1 and demonstrates that a significant number of journals publish only a very few articles on bibliometric analysis during this period.

The number of citations per article for the whole period varies slightly. The data for tier 1 is around 30 % larger than that for tier 4 and is in accordance with the similar accumulated citations of all types of articles published in journals such as “*Scientometrics*”, “The citation rate is markedly higher for the methodical, theoretical studies (tier 2) but more equal for the applied studies irrespective of publication channel. We observe again that the citing articles, irrespective of the cited items, are published in a significant number of non-ILS journals but in far fewer “library” journals.

It is evident from Table 3 that articles published in non-ILS journals, tier 4, are preferentially more likely to be cited in journals from the same category. In fact, the number of non-ILS (tier 4) publications is 1.7 times larger than the number of ILS publications. However, the citation factor, i.e. the number of citing articles, between the two categories, when we deal with the same non-ILS (tier 4) publications, is about 4.8. When we deal with the “subject” related studies (tier 3) publications, it is the other way around and the ILS citation factor is 1.5 times higher than the non-ILS factor. This data certainly illustrates the citation advantage of publishing within the relevant category and targeting the more professional readers. The literature is preferentially cited by users belonging to the same community.

In contrast, the citation pattern is not very different depending on whether the article is marked as theoretical or applied as long as it is categorized as ILS.

**Fig. 2:**
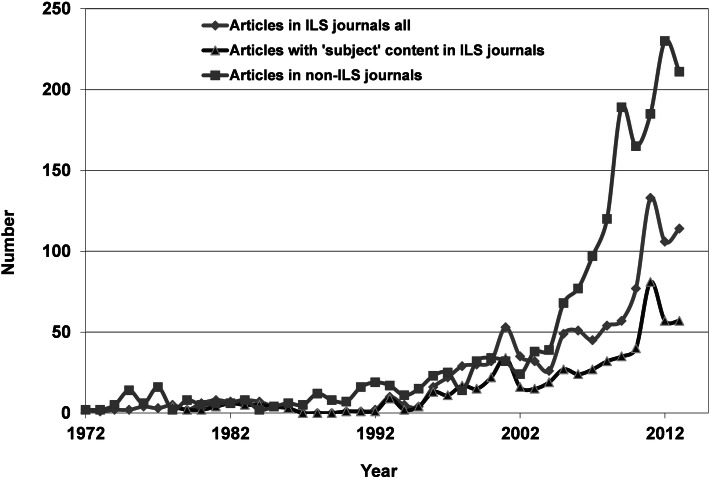
Total number of articles on bibliometric analysis published in *WoS* core journals

The time analysis for the publications within tiers 1, 3 and 4 is demonstrated in Fig. 2.

**Fig. 3:**
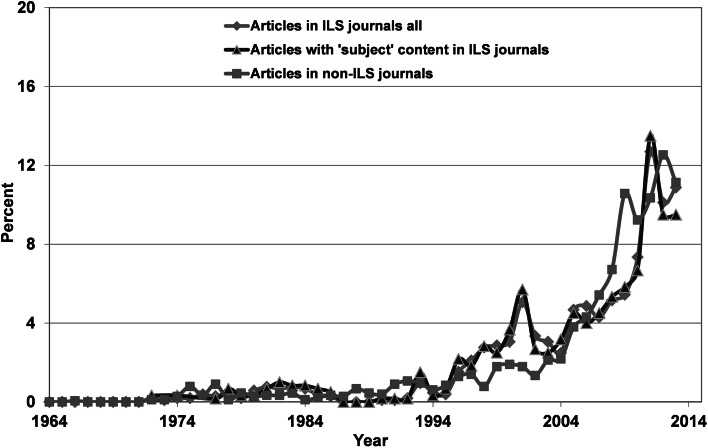
Relative number of articles on bibliometric analysis published in *WoS* core journals

It can be seen that very few instances of bibliometric analysis were carried out before 1994, and they were mainly published in non-ILS journals. From 1994 onwards, the number of analyses rose almost exponentially within all three tiers with the following correlation coefficients (ILS-articles: *r*^2^ = 0.78, ILS articles with “subject” content: *r*^2^ = 0.75, non-ILS articles: *r*^2^ = 0.93). The rising trend apparently levels out during the last couple of years. If we consider the relative numbers in Fig. 3, it is clearly seen that during the decade 1994–2004, relatively more articles are published in “library” journals compared to non-ILS journals. In the years from 2005 up to 2010, the relative number of publications in non-ILS journals increased, which may indicate the usefulness of publishing in journals targeting the primary users of these investigations.

### Citation impact

**Fig. 4:**
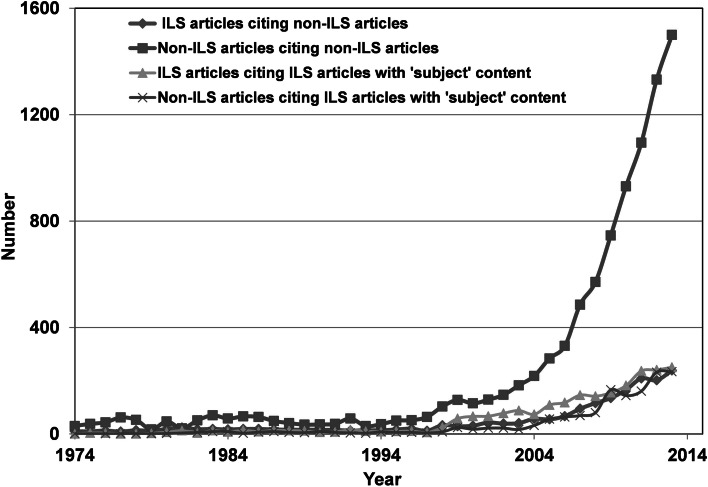
Total numbers of articles which cite articles on bibliometric analysis published in different *WoS* journals

When the tendency in the number of citations of the publications in tier 3 and 4 is considered (Fig. 4), a progression can be seen in the same manner as in Fig. 2, e.g. the rise in non-ILS articles citing non-ILS articles since 1994 shows an almost exponential growth (*r*^2^ = 0.99) The absolute number of citations of non-ILS articles obtained from non-LIS articles is significantly greater than from ILS-articles. Until about 2010, the numbers increased in the same manner but recently, the numbers of ILS citations seems to reach a steady level. ILS citations of ILS articles with “subject” content seem to dominate until 2008. After this period, an increase can be seen in non-ILS citations compared to “library” citations. It can probably be explained by a slower awareness of bibliometric methods in the community outside the information professionals. In contrast, the tendency in the relative number of citations of the tier 4 publications from both communities progressed in a more comparable fashion.

**Fig. 5:**
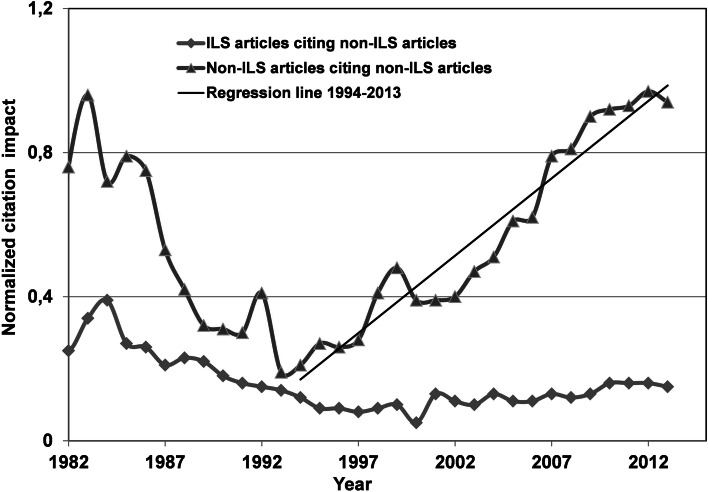
Normalized citation impact according to Eq. 1 for non-LIS articles on bibliometric analysis. Regression line: *r* ^2^ = 0.93

If we consider instead the normalized citation impact of non-ILS articles calculated from Eq. 1 (Fig. 5), it is evident that the interest in bibliometric analysis reached an all-time low in 1994. After this period, the normalized citation impact of non-ILS articles increased in an almost linear fashion (*r*^2^ = 0.93). The same rate, in case of ILS articles, remained almost constant after 1994.

**Fig. 6:**
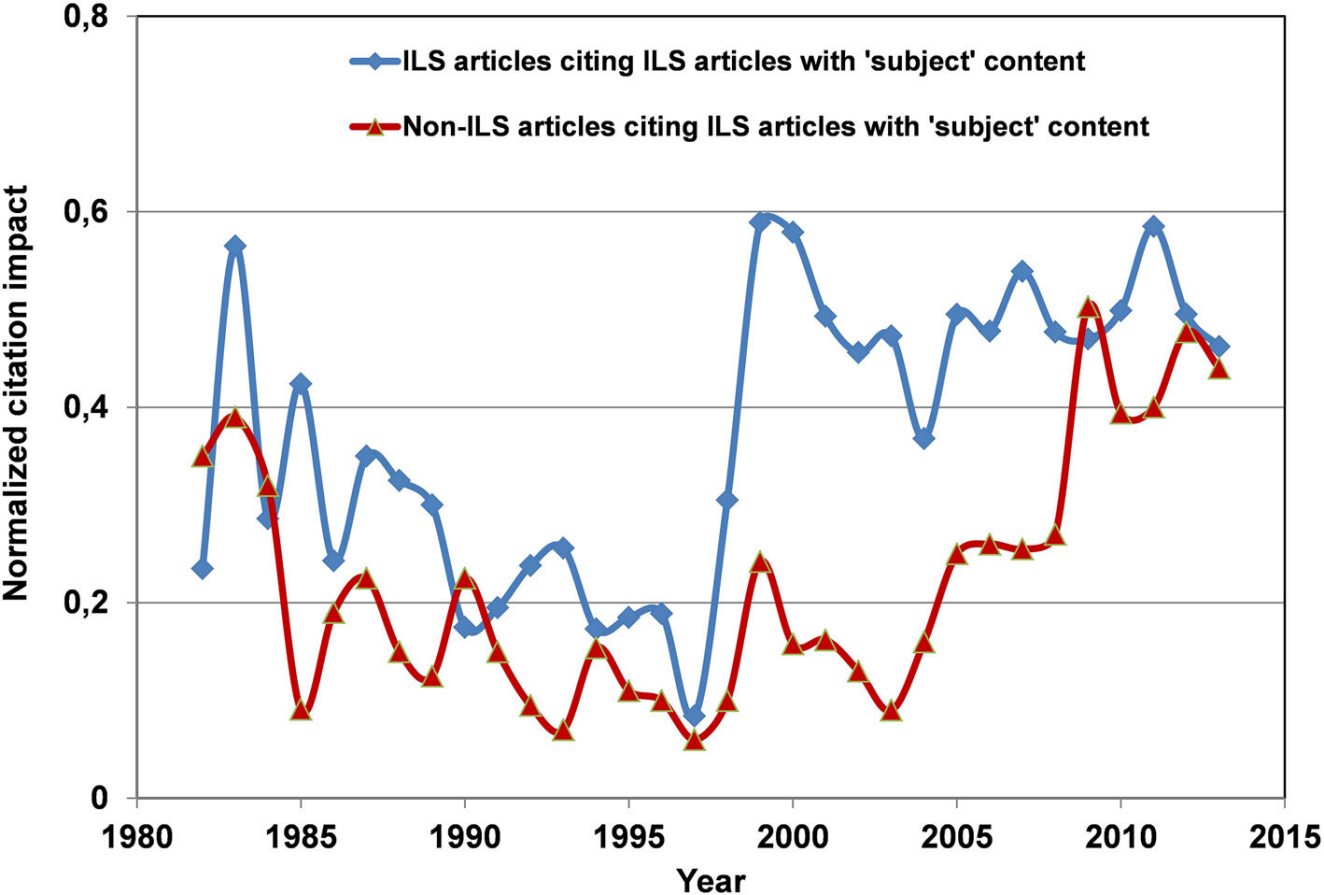
Normalized citation impact according to Eq. 1 for ILS articles with subject content on bibliometric analysis

The normalized citation data from the articles in tier 3 is shown in Fig. 6. The scattering of the data, especially during the first period, is due to poor statistics and can be observed in the first part of Fig. 5 as well. The largest impact is found in case of the ILS citations and this result deviates from the tier 4 data shown in Fig. 5. In the most recent period, the measured impact in the ILS and non-ILS community seems to become similar. Again, we observe an all-time low in the normalized impact, albeit a few years later, around 1997. This trend is most pronounced in case of ILS citations which jump to an almost constant high level in a matter of 2 years after a period of steady decline. On the other hand, the non-ILS citations increase at a more constant rate after 1997.

### Country analysis

The way in which publications and citations are distributed among those countries which contribute the most to the field during the time period 1964–2013 within tier 4 (Tables 4, 5 will now be investigated.

**Table 4:** Articles that refer to bibliometric analysis published between 1964 and 2013 and classified by *WoS* within the non-ILS category (tier 4). Distribution by country and year

No.	Country	Number of articles published between						(2002–2013)/(1964–2013) (%)	Mean citation rate 2011–2013^a^	Mean citation rate 1964–2013^a^
		2011–2013	2008–2010	2005–2007	2002–2004	2002–2013	1964–2013			
1	USA	140	95	81	41	357	530	67.4	2.9	14.5
2	PRC (China)	113	50	11	4	178	183	97.2	1.6	2.5
3	Spain	47	47	29	5	128	151	84.8	1.8	5.2
4	England	32	33	14	9	88	111	79.2	3.6	11.3
5	Taiwan	60	31	12	0	103	104	99.0	2.8	4.9
6	Germany	32	38	17	5	92	96	95.8	2.9	6.0
7	Canada	34	29	11	4	78	89	87.6	2.8	9.1
8	Australia	28	28	6	2	64	73	87.7	2.2	5.1
9	Italy	23	14	5	6	48	55	87.2	5.3	9.5
10	Netherland	13	11	11	1	36	46	78.3	2.9	14.8
No.1-10, all		449	338	176	73	1036	1292	80.2	2.3	9.4
All countries		626	474	242	101	1443	1806	79.9	2.3	9.1
No.1-10, all/all countries(%)		71.7	71.3	72.7	72.3	71.8	71.5	–	100.9	103.2

^a^Based on number of citations to articles published by the country in the same period

**Table 5:** Articles that cite the articles on bibliometric analysis which are published between 1964 and 2013 and classified by *WoS* within the non-ILS category (tier 4). Distribution by country

No.	Country	Citing articles^a^ 1964–2013	Citing articles, % of all countries	Citing articles/no. of articles1964–2013
1	USA	4075	35.8	7.7
2	PRC (China)	724	6.4	4.0
3	Spain	752	6.6	5.0
4	England	1041	9.2	9.4
5	Taiwan	342	3.0	3.3
6	Germany	650	5.7	6.8
7	Canada	647	5.7	7.4
8	Australia	462	4.1	6.4
9	Italy	426	3.7	7.7
10	Netherland	496	4.4	10.8
“No.1-10”, all		8433	74.2	6.6
All countries		11,372	–	6.3

^**a**^Based on number of articles citing *all* articles that refer to bibliometric analysis and published during 1964–2013 in the non-ILS category

The dominating country in the field, with the most publications, is USA but the three countries PRC (China), Taiwan and Germany have also published an increasing number of works in the field during the latest decade. Especially PRC is now at a comparable level to USA. In contrast to this, the citation rate of the PRC articles is relatively low, but improving, when compared to the other countries in the table. Spain can be seen to have a strong tradition within the bibliometric field, and publications from The Netherlands are also cited well above the average. The mean citation rate of the newest articles (2011–2013) between the top-10 countries in tier 4 is not significantly different. In contrast, when the whole 50 years period is taken into account, countries such as USA, England and The Netherlands have the largest impact of their article production. The last column (Table 5) shows that the countries with the highest citation rates of their own productions also tend to be those which cite the production of other scholars most often.

The data within tier 3 during the period 1964–2013 (Tables 6, 7) shows no major difference from the data in tier 4 although 3 new countries, namely Belgium, India and South Korea enter the top-10 list of the most productive countries. Something to note is the exorbitantly high mean citation rate of the articles published by authors from USA, The Netherlands and Belgium. Apparently, the publications, with contributions from these countries, are particularly valued in the ILS community.

**Table 6:** Articles that refer to bibliometric analysis and are classified as “Library and Information Science” with “subject” related studies (tier 3). 1964–2013. Distribution by country and year

No.	Country	Number of articles published between						(2002–2013)/(1964–2013) (%)	Mean citation rate 2011–2013^a^	Mean citation rate 1964–2013^a^
		2011–2013	2008–2010	2005–2007	2002–2004	2002–2013	1964–2013			
1	USA	29	13	13	10	65	122	53.2	2.7	16.2
2	PRC(China)	44	18	7	2	71	76	93.4	1.8	5.6
3	England	19	11	7	9	46	56	82.1	1.5	11.3
4	Taiwan	31	13	5	5	54	55	98.2	1.1	8.1
5	Spain	13	12	6	4	35	42	83.3	2.0	4.5
6	India	5	4	3	7	19	30	63.3	1.4	7.7
7	Netherland	7	6	2	3	18	26	69.2	2.8	15.3
8	Belgium	6	3	6	5	20	23	87.0	0.7	13.2
9	Germany	6	6	3	0	15	19	78.9	2.7	11.3
10	South Korea	7	6	4	0	17	19	89.5	2.4	7.1
No.1-10, all		139	80	53	38	310	414	74.5	1.7	10.7
All countries		195	107	78	50	430	600	71.7	1.7	9.4
No.1-10, all/all countries (%)		71.3	74.8	67.9	76.0	72.1	69.0	–	–	–

^a^Based on number of citations to articles published by the country in the same period

**Table 7:** Articles that cite the articles on bibliometric analysis which are classified as “Library and Information Science” with “subject” related content (tier 3). 1964–2013. Distribution by country

No.	Country	Citing articles^a^ 1964–2013	Citing articles, % of all countries	Citing articles/no. of articles 1964–2013
1	USA	963	26.1	7.9
2	PRC(China)	302	8.2	4.0
3	England	382	10.3	6.8
4	Taiwan	206	5.6	3.7
5	Spain	312	8.4	7.4
6	India	98	2.7	3.3
7	Netherland	183	5.0	7.0
8	Belgium	118	3.2	5.1
9	Germany	155	4.2	8.2
10	South Korea	114	3.1	6.0
“No.1-10”, all		2519	68.2	6.1
All countries		3692	–	6.2

^a^Based on number of articles citing *all* articles that refer to bibliometric analysis, published during 1964–2013, and classified as “Library and Information Science” with “subject” related content

There is also a tendency among the up-and-coming countries PRC, India and Taiwan to publish their results more often in tier 3 journals. The impact or mean citation rate of the “subject” articles published in the ILS category shows a greater scattering than the tier 4 data during the periods investigated. They range from rather low impact in the case of the up-and-coming countries to a high impact in the case of USA and The Netherlands.

The amount of citing articles of the whole production within the tier (Table 7) does not reflect the number of citations obtained by the individual countries to the same extent as the data for tier 4.

### Subject analysis

The articles in tier 4 have been divided into six main subject categories. The numbers of publications as well as their citation rates are listed in Table 8.

**Table 8:** Articles (tier 4) that refer to bibliometric analysis classified in major *WoS* non-ILS subject-categories. Published 1964–2013 and cited during the same period

Subject category	Number of articles	Times cited	Citing articles	Citing articles not classified in tier 1 (% of all)	Citations per article
Multi disciplinary Science	126	2130	2483	1718 (69.2)	16.9
Life Science	97	1035	898	718 (80.0)	10.7
Health Science	658	5826	4005	3489 (87.1)	8.9
Applied Science(Engineering materials)	182	1332	1009	853 (84.5)	7.3
Physical Science (Physics, Chemistry, Mathematics etc.)	109	708	633	513 (81.0)	6.4
Computer Science	175	593	507	315 (62.1)	3.4

There are a significant number of publications within each category, which confirms that bibliometric analysis is used as a tool in all scientific communities. Generally, bibliometric analysis of multidisciplinary science has the highest number of citations per article. Health science, due to its many sub-disciplines, has the highest number of publications but also some of the highest citation rates. In contrast, computer science is notably less cited than the other subject areas. The articles are, to a very large extent, cited by the scientific communities themselves but computer science has a relatively larger number of citations from the library and information field. In contrast, health science has the highest share of bibliometric investigations within the community itself.

A further analysis of keywords in the publications of tier 4 (Table 9) demonstrates that medicine, with its many sub disciplines, is a major object of bibliometric analysis. Management, business and operation research are extensively studied as is computer and information science in its various guises. The list proves that a large number of subjects and trends are represented and shows the widespread use of bibliometric analysis as a tool for documentation within the different communities.

**Table 9:** The 40 most frequent, non-trivial keywords which occur in tier 4, non-ILS references on bibliometric analysis. *WoS*. 1964–2013

Keyword (subject)	Occurrence
Management	162
Computer science. Information systems	114
Medicine. general and internal	87
Computer science. Artificial intelligence	81
Engineering. Industrial	79
Operations research and management science	72
Healthcare sciences and services	65
Surgery	64
Business	63
Medicine	62
Neurosciences	60
Environmental sciences	60
Computer science	56
Psychiatry	56
Chemistry. Multidisciplinary	54
Nursing	54
Computer science. Interdisciplinary applications	52
Public. Environmental and occupational health	52
Clinical neurology	52
Engineering. Electrical and electronic	51
Health	43
Biology	41
Innovation	41
Ecology	39
Economics	38
Technology	38
Medical informatics	38
Social sciences. Interdisciplinary	38
Education. Scientific disciplines	37
Psychology	36
Computer science. Information	36
Oncology	35
Anesthesiology	34
Rehabilitation	34
Ophthalmology	34
Computer science. Theory	33
Cell biology	32
Engineering. Multidisciplinary	31
Operations research and management	28
Pharmacology and pharmacy	28

## Discussion and conclusion

The data in the present analysis was tentatively divided into two groups in order to discriminate between the fundamental studies of bibliometric analysis concerning theoretical issues and the more applied studies. This was done for both the studies and the citing articles. It is also evident by assigning different codes or descriptors to the ILS-articles that a large fraction can be classified as “subject” based studies and must be placed in its own subcategory. This is another division of the literature than used by Jonkers and Derrick (2012) who dealt with multidisciplinary science as a separate classification and analyzed it as a non-ILS subcategory. In this way multidisciplinary studies are represented in ILS as well as non-ILS literature and the analysis in the two groups complement each other. The data concerning the absolute number of articles in the ILS and the non-ILS category is comparable to the almost exponential growth recorded during the last two decades by Jonkers and Derrick (2012). They found a substantial increase in the number of publications about development and improvement of bibliometric indicators as well as methods during recent years in both the ILS category and, although to a lesser extent, the non-ILS category. In the present data, the fundamental studies (tier 2) are published in a small number of “Information and Library Science” journals with *Scientometrics* and *Journal of the American Society for Information Science and Technology (JASIST)* as the leading publishing channel. A large number of “applied” studies are still published in the “Information and Library Science” journals (tier 3), which accounts for about 2/3 of the articles. The journal *Scientometrics*, established in 1978, published 60 % of all the applied studies in this group while the number in case of the theoretical studies (tier 2) is only 45 %. The same numbers in case of *JASIST* are 17 and 8 % percent respectively which makes *Scientometrics* the leading journal for applied bibliometric studies.

The applied non-ILS based studies in tier 4 are found in a much larger number of different “subject” oriented journals, which reflects the multidisciplinary relevance of these articles for the professional communities.

Our time-lapse data further shows that there are relatively few articles on bibliometric analysis before 1994. It is almost as if there was a threshold for this type of publications and it is probably due to a number of factors: Firstly, the need for a sufficient volume of materials to become analyzed and second, the advancement of computerized methods for data treatment and the general availability of electronic versions of well-established databases such as *SCI*. The emergence and widespread distribution of the internet and the *World Wide Web* within the scientific communities also made data gathering easier. Of course, the general awareness and hence demand for these types of analysis plays a significant role.

The studies published in the non-ILS category grew more dominant through this period probably due to more focus on research performance evaluation, while the number of theoretical studies was relatively constant. This is in spite of the fact that a large number of articles on different modifications of bibliometric indicators have emerged in the wake of the paper about the h-index published by Hirsch (2005). These studies are most likely to be found among the tier 2 articles.

The citation rate is not very different for the articles in the four different tiers investigated here, although theoretical studies, indexed in the ILS category, tend to be the most cited. This result is in accordance with the study of Jonkers and Derrick (2012) who use field-normalized citation data. One could indeed predict and expect reasonably lower citation rates for the “subject” based types of analysis. These studies are more suited for a smaller professional community within the field, not a general audience. The possibility exists that the practitioners in the fields which are targeted by non-ILS publications are less likely to make literature contributions that appreciate bibliometric studies of the field via citations (Derrick et al. 2012). One could simply state that to this community, it is the results of the bibliometric analysis which is the most important and not the analytical process itself. Of course, it certainly places a greater responsibility on the ILS-community to make sure that the tools and methods of bibliometrics are adequately described and available for use.

The above pattern is reflected again in the number of citing articles and journals. The number of citing articles per journal is very large for ILS articles cited in the same category. The articles classified in the non-ILS category are still cited in many journals but by far the largest number of citing journals is also found in the non-ILS category. This shows that articles that refer to bibliometric analysis are not only scattered around in many “subject” based journals but are cited with the same frequency in the similar type of journals as well. The applied studies in tier 3 are cited more equally in the ILS and non-ILS articles. From 1994 onwards, library citations dominated but from around 2008, the non-ILS citations caught up. This illustrates the importance of choosing your publication channel. There are relatively more citations within the same category. The peers of the authors of a publication expect to read, publish and cite in similar journals.

On the other hand this statement could be seen in a different light when we consider the theoretical or methodical non-ILS articles in tier 2. They are cited in almost the same manner as the applied articles. Apparently, the methodical studies are well noticed by the non-ILS community as well.

If we look at the absolute number of articles that cite articles in the non-ILS category, citations within the same category are dominating. This trend becomes clear from 1994- onwards. The number of citations from “subject” articles is also larger up to 1994, however, most citations are found in the journal *Current Contents*. During this period, many columns in this weekly journal treated the fundamental, intrinsic problems with the use of the bibliometric analysis as a working tool. Up to the year 1992, 439 out of 898, representing almost half of all citations, are registered in *Current Contents*. The journal could reasonably be placed in the ILS-category but the division used by *WoS* in the earlier work of Jonkers and Derrick (2012) was maintained.

The normalized data indicates a major difference between the ILS and non-ILS field. While the impact of the non-ILS publications continues to rise at a steady pace within the community itself, the impact is more constant in the ILS community. Obviously, the application of bibliometric methods becomes more and more accepted by the community who were the original target of these methods, and it is honored via citations. Similar publications in ILS journals receive rising awareness from both the ILS and the non-ILS community. This last finding is important because the development of the bibliometric field will benefit from a closer interrelationship between the groups, especially in times when new metrics and indicators enter the field. Indeed, the data, especially in tier 3, indicates interrelations between the ILS and non-ILS communities and increased participation of bibliometricians in tier 4 publications could further professionalize the field. The whole science of bibliometry could indeed benefit from border crossing between the applied and non-applied fields. Bibliometricians could demonstrate the methods available in a practical context and in the same manner, the co-operation with the professional, “subject” orientated communities could improve the theoretical development of the field. The normalized citation data for both tier 3 and tier 4 reached an all-time low around 1997 and 1994, respectively. Obviously, apart from the scarce data, both tiers became less cited up to the period when bibliometric tools and data became available on-line. The following increase is clearly correlated to the similar increase in the number of articles about bibliometric analysis observed around the same time period for both tiers as shown in Fig. 2. Actually, the increase in number of publication in tier 3 lags behind the data of tier 4. This could explain the similar 3-year lag in the normalized citation data.

Next, we turn to the subjects studied. The use, publication and citation pattern are similar for the applied, health, physical and life sciences. Computer science has a lower impact within the community. This could, in itself, be due to the fact that this field is regarded as fast moving and therefore its literature is seen as having a shorter lifespan.

By far the highest citation rate is gained by publishing bibliometric analyses of multidisciplinary studies. This can, of course, be explained by the fact that the sheer number of people involved is higher and probably, according to *InCites.Essential Science Indicators* (2015) in a higher impact of multidisciplinary studies itself, but the assumed beneficial effects of cooperation between scientific groups apparently also show up in the bibliometric analysis of this literature type. We believe this issue deserves more attention.

Our findings show clearly how a number of countries have dominated the field of bibliometric analysis and still partially do. USA, PRC and England are the leading countries but the number of articles published by authors from Spain and The Netherlands is also noteworthy. The articles from the latter country are not very numerous but their impact is far greater. As an example, the *Centre for Science and Technology Studies* in Leiden, established in 1989, is a well-reputed institution for fundamental bibliometric research. It is well known that country indicators are sensitive to the delimitation of journals included in the investigation, and the leading countries are particularly present in high-impact journals (Zitt et al. 2003).

In general, the articles from the most productive countries are among the most cited. On the other hand, this tendency seems to change with the growing publication rates from up-and-coming countries such as PRC, Taiwan and India. It probably reflects the domains investigated by these countries. We could ask whether they to a greater extent treat subjects that have roots within the local communities? The time it takes for a scientific discipline to become fully established could also play a role so that more established fields may be favored. This tendency is confirmed when we consider the number of articles from the various countries citing the pool of articles from all countries. The ratio between citing articles and own production is relatively low for the upcoming countries. Furthermore, if we compare this ratio with the mean citation rate of the articles from individual countries, it is evident how they are correlated. Countries which have a highly cited production are also among the most citing countries themselves. It points towards a self-perpetuating process of giving and taking of references in the field of bibliometric analysis. It can be stated that the most well established groups or countries are dominating or leading the field and this shows up in the citation patterns.

The subjects investigated and the aim of the analysis varies considerably, as indicated in the list of keywords downloaded from the publications. The list indicates clearly that the bibliometric analysis has become a mature analytic tool and is widespread in many contexts. This is not pursued in great detail in the present work; however it would be of interest to consider the individual types of analysis that are carried out as a target for further research. Are bibliometric analyses in some specific subject areas more frequently cited than others? Do the up-and-coming countries primarily investigate, via the use of “bibliometric methods”, subjects in the literature related to “local” issues or do they have a more “global” perspective? This question has been partly covered in a recent bibliometric work by Tang (2013) although this deals with the matter on a more general, subject specific basis. When it comes to citations, a more detailed analysis may be useful. Which categories of readers actually cite the publications? The data in the “library and information science” category suggest that these types of analysis can reach a wider audience and not just the professionals within the relevant field itself. The number of publications about bibliometric analysis has now reached a sufficient level for many of these of questions to be answered in a reliable way.

A related question concerns the impact of bibliometric analysis and a possible correlation with the impact of the literature about the objects actually studied. In fact, these types of correlation analysis could indicate whether some areas are underrepresented from a bibliometric point of view. Does the bibliometric analysis of high-impact subject fields receive sufficient attention from its intended audience? Such types of analysis could be useful for the bibliometric studies of both larger and smaller subject areas. We could, as an example, consider the mean impact of the bibliometric analysis of the entire subject categories shown in Table 8 and compare it to similar impact data from the subjects itself. We will pursue this in further detail in a forthcoming investigation, but a tentative look at data from *InCites. Essential Science Indicators* (2015) may point at a close correlation.

Finally, we can conclude that the number of publications using the bibliometric analysis as a tool for science studies has been rising steadily during recent years. This can be due to a number of factors: A sufficient number of articles need to be published within a field in order to evoke a bibliometric investigation, and the tools available to treat large data sets are now widely in use. An increased demand for these types of analysis in evaluation of research and productivity is likely within many scientific communities, by politicians as well as funding agencies. Bibliometric analysis has apparently been seen as a valuable method for evaluating scientific production and it has a rising impact especially in the non-LIS community. The present work demonstrates how bibliometric analysis is gradually becoming accepted as a useful tool for the professional community and not just an academic tool for bibliometricians.
